# The impact of nurse staffing levels and nurse’s education on patient mortality in medical and surgical wards: an observational multicentre study

**DOI:** 10.1186/s12913-019-4688-7

**Published:** 2019-11-21

**Authors:** Filip Haegdorens, Peter Van Bogaert, Koen De Meester, Koenraad G. Monsieurs

**Affiliations:** 1 Centre for Research and Innovation in Care (CRIC), Department of Nursing and Midwifery Sciences, University of Antwerp, Universiteitsplein 1, 2610 Wilrijk, Belgium; 2 Department of emergency medicine, Antwerp University Hospital, University of Antwerp, Wilrijkstraat 10, 2650 Edegem, Belgium

**Keywords:** Mortality, Nurse staffing, Nurse education, Outcomes

## Abstract

**Background:**

Growing evidence indicates that improved nurse staffing in acute hospitals is associated with lower hospital mortality. Current research is limited to studies using hospital level data or without proper adjustment for confounders which makes the translation to practice difficult.

**Method:**

In this observational study we analysed retrospectively the control group of a stepped wedge randomised controlled trial concerning 14 medical and 14 surgical wards in seven Belgian hospitals. All patients admitted to these wards during the control period were included in this study. Pregnant patients or children below 17 years of age were excluded. In all patients, we collected age, crude ward mortality, unexpected death, cardiac arrest with Cardiopulmonary Resuscitation (CPR), and unplanned admission to the Intensive Care Unit (ICU). A composite mortality measure was constructed including unexpected death and death up to 72 h after cardiac arrest with CPR or unplanned ICU admission. Every 4 months we obtained, from 30 consecutive patient admissions across all wards, the Charlson comorbidity index. The amount of nursing hours per patient days (NHPPD) were calculated every day for 15 days, once every 4 months. Data were aggregated to the ward level resulting in 68 estimates across wards and time. Linear mixed models were used since they are most appropriate in case of clustered and repeated measures data.

**Results:**

The unexpected death rate was 1.80 per 1000 patients. Up to 0.76 per 1000 patients died after CPR and 0.62 per 1000 patients died after unplanned admission to the ICU. The mean composite mortality was 3.18 per 1000 patients. The mean NHPPD and proportion of nurse Bachelor hours were respectively 2.48 and 0.59. We found a negative association between the nursing hours per patient day and the composite mortality rate adjusted for possible confounders (B = − 2.771, *p* = 0.002). The proportion of nurse Bachelor hours was negatively correlated with the composite mortality rate in the same analysis (B = − 8.845, *p* = 0.023). Using the regression equation, we calculated theoretically optimal NHPPDs.

**Conclusions:**

This study confirms the association between higher nurse staffing levels and lower patient mortality controlled for relevant confounders.

## Background

Nurses represent the largest group of hospital workers and are responsible for the majority of patient care. Investigating the impact of their work environment and staffing on the quality of healthcare and patient safety is therefore crucial. There is a growing body of evidence indicating that inadequate nurse staffing in acute care hospitals is associated with adverse events such as patient falls, health-care related infections, medication errors and in-hospital mortality [1, 2]. Aiken et al. showed that improved nurse staffing and a higher proportion of nurses with a bachelor’s degree reduced the likelihood of a hospitalised patient dying within 30 days of admission [3]. It has been hypothesised that adequate nurse staffing influences the quality of patient surveillance because it allows nurses to spend more time in direct care. Insufficient staffing leads to the rationing of time to care, which has an important impact on the occurrence of missed care [4]. This could be an explanatory factor linking nurse staffing levels and patient outcomes such as in-hospital death [5]. However, it is unclear how nursing workload should be measured and how wards should be staffed to provide safe care. Ideally, hospital management aims for a balanced state between workload and the number of nurses staffed. Patient classification systems (PCSs) were developed to guide the allocation of hospital resources and are used by governmental organisations to determine hospital financing [6]. PCSs include objective and subjective measures of patient needs and other care dimensions but are difficult to measure and are insufficiently backed by evidence [7]. Nurse-to-Patient Ratios (NPRs) are defined as the proportion of nurses available per patient and are used to distribute the available nursing staff across hospital wards [8]. However, NPRs do not take into account the complexity of care or ward characteristics. NPRs estimate the number of nurses needed considering the amount of occupied beds on a specific ward. The nursing hours per patient day (NHPPD) method is a commonly used NPR which is calculated by dividing the available nursing hours by the number of admitted patients during a 24-h period. Policy makers have been using PCSs to estimate ward acuity and NPRs to determine minimal nurse staffing levels for each ward [9]. To date, it is not clear which NPR is suitable considering patient acuity and clinical outcome targets [10]. Because of the overwhelming evidence linking nurse staffing with patient outcomes, there has been considerable academic discussion about ward based mandatory staffing levels [11]. Mandatory nurse staffing levels are in place in the state of California (United States) and in Victoria and Queensland (Australia) [9, 12]. However, since no evidence-based nurse staffing guidelines exist to date, most governments and healthcare organisations are hesitant to support a mandated ward-based minimum. Furthermore, hospital managers and nurse leaders continue to struggle on a daily basis when allocating available nurses to hospital wards taking into account patient acuity and outcomes, nurse’s experience and education level, ward workflow and financial factors [10]. It is reasonable to hypothesize that if there were more nurses available with an appropriate skill-mix, patient care would improve. However, the most important limitation in current research is that the majority of studies investigating nurse staffing and patient outcomes use aggregated hospital level data making the translation to the ward level impossible. Additionally, it remains unclear which methodology is appropriate when measuring nurse staffing and workload [13]. The objective of this study was to explore the relation between nurse staffing levels and patient mortality on medical and surgical wards considering nurse’s education level, team composition, patient’s age and comorbidity.

## Methods

### Study design and participants

This was an observational longitudinal study retrospectively analysing the control group of a stepped wedge randomised controlled trial (SW-RCT) concerning 14 medical and 14 surgical wards in seven Belgian hospitals [14]. In the original study, we investigated the effect of a rapid response system on patient outcomes. Therefore, we collected data on patient morbidity and nurse staffing levels since we hypothesised that they could be important confounders. All patients admitted to the study wards during the control period from October 2013 to January 2015 were included in this study. Pregnant patients or children below 17 years of age were excluded because, in the SW-RCT, the intervention was not suitable for these patients. Approval of the ethics committees of all local hospitals was obtained beforehand (registration number: B300201317835). This study was carried out in seven hospitals geographically spread throughout Belgium (Flanders, Brussels and Wallonia). The SW-RCT study protocol, including the approach to collecting data, was described in an earlier publication [14]. In the control group 34,267 patient admissions were included during four periods of 4 months each. Because of the nature of the stepped wedge design in the original study, wards and hospitals had unequal sample sizes.

### Measures

We collected data on two levels during the 16-month study period: the patient level and the ward level. Patient level data were collected in all admitted patients on the study wards. Ward level data were collected using a subsample of patients (comorbidity) and ward staff records (all staffing measures).

### Outcomes

All primary and secondary outcomes in this study adhered to the patient level. The primary outcomes were: crude mortality, unexpected death, death until 72 h after Cardiopulmonary Resuscitation (CPR) and death until 72 h after unplanned Intensive Care Unit (ICU) admission. Crude mortality and unexpected death concerned a patient’s death while admitted to a study ward. Unexpected death was defined as death without a do not resuscitate (DNR) order or, without palliative or terminal care or, without family attending during the process of dying or, without cessation or limiting of active therapy in untreatable disease. Additionally, a composite outcome (combined mortality rate) comprising unexpected death, death until 72 h after CPR and death until 72 h after unplanned ICU admission was constructed to include patients who received CPR or were transferred to the ICU but could not be rescued. The rationale was that this composite outcome, which is an estimate for the adverse mortality, should be sensitive for the occurrence of missed care (i.e., there is an increased risk of death when nurses fail to detect deterioration and patients are transferred too late to the ICU). The secondary outcomes in this study were: CPR, unplanned ICU admission and hospital length of stay (LOS). The outcomes used in this study were defined in our earlier publication [14].

### Patient characteristics

We collected the age of all admitted patients because it is an important explanatory factor of in-hospital death [15]. To estimate the ward’s acuity level, we calculated the Charlson Comorbidity Index (CCI) in 30 consecutive patient admissions across all wards starting on the last Monday of the second month of each period (T0-T3) [16]. Hospitals were blinded for the collection dates of all comorbidity data.

### Staffing and education

In this study we collected staffing measures per individual study ward. In Belgium, there are two different nurse educational levels: the bachelor’s degree nurse (European Qualification Framework, EQF level 6) and the certificate nurse (EQF level 5) [17]. Nurses with a bachelor’s degree followed a 3 year, scientifically orientated programme after graduating from secondary school. Certificate nurses undertook a predominantly practical nurse training of 3 years in the fourth grade of secondary school. The total nurse staffing levels were calculated using government mandatory registration data and included both types of nurses. The daily staffing levels, expressed in hours, were available for 15 days in each period (T0-T3). Nursing hours per patient day (NHPPD) were calculated for each of those 15 days per period by dividing the total amount of available nursing hours by the number of admitted patients. To estimate nurse’s education level, we calculated the proportion between the nursing hours with a Bachelor educational level attained and the total amount of nursing hours per 24 h. Nurses are supported in their daily activities by other healthcare workers who could have a significant impact on the experienced workload and the nurse work environment. Therefore, the available hours per patient day were calculated in the same manner as mentioned before for nurse assistants (EQF level 4) and the logistic support staff (EQF level 4). Nurse assistants are supervised by nurses and assist in direct patient care such as bathing patients or helping with toilet visits. Moreover, they can take care of a limited amount of nursing activities (e.g. measuring vital signs or administering medication prepared by a nurse). Logistic support personnel are hospital ancillary workers who support nurses and nurse assistants in the care they provide (e.g. transferring patients under supervision, providing meals or ward stock replenishment).

### Statistical analysis

Before analysis, data were aggregated per ward and per period resulting in a total of 68 measurements. Only data concerning the control group were included in this study explaining the unequal amount of measurements per hospital and per period. The age, CCI, LOS and all staffing measures were averaged, and all study outcomes were expressed as proportions (events per 1000 admissions).

Analyses were performed using IBM SPSS Statistics version 24 for MAC OS. Ward level data were compared between wards or hospitals using a Kruskal Wallis test because of the relatively small sample size (*n* = 68 estimates). To investigate the relation between nurse staffing levels and outcome variables, linear mixed models (LMM’s) were fitted. A linear mixed model analysis is the most appropriate approach when dealing with repeated measures at different timepoints (multiple measures per ward) and in case of data clustering (multiple wards belonging to one hospital) [18]. Moreover, LMM’s are more reliable than other strategies for analysing repeated measures data when dealing with missing and unbalanced data [19]. Two different models were fitted using the MIXED procedure in SPSS, both including clustering as a random effect (ward nested in hospital) and study time as a fixed effect (study period T0-T3). The Aikake Information Criterion (AIC) combined with the Schwarz’s Bayesian Information Criterion (BIC) were used to compare model fit. Model one showed the relation between nurse staffing levels and all study outcomes adjusted for clustering and study time. Model two was based on model one but also adjusted for the proportion of nurse Bachelor hours, nurse assistant HPPD, logistic support staff HPPD, CCI and patient’s age. Furthermore, we estimated the theoretically optimal NHPPD per ward using the linear regression equation resulting from model two with the combined mortality rate as dependent variable. The linear regression equation (y = (a*×^1^) + (b*×^2^) + (c*×^3^) + (d*×^4^) + (e*×^5^) + (f*×^6^) + intercept) was rearranged, assuming a combined mortality rate of zero (y = 0) and (×6 = NHPPD estimate), into: NHPPD^optimal^ (f) = ((− 1*intercept) - (a*age estimate) - (b*CCI estimate) - (c*proportion bachelor hours estimate) - (d*nurse assistant HPPD estimate) - (e*logistic support staff HPPD estimate)) / NHPPD estimate.

## Results

We aggregated data from 34,267 patient admissions resulting in estimates per ward and per period concerning patient’s age, length of stay and the proportion of patients who reached one of the study outcomes. Additionally, we aggregated all comorbidity data of 1860 patients (T0: 840, T1: 660, T2: 300 and T3: 60). Hospital seven had missing data concerning comorbidity in T2 and T3. Lastly, staffing data of 1020 days (T0: 420, T1: 330, T2: 210 and T3: 60) were aggregated to the ward level. Aggregating all data to the ward level and per period resulted in 68 estimates of seven hospitals, 28 wards and over four periods (Table 1). Because of the nature of the original control group in the stepped wedge randomised controlled trial, estimates across time and between hospitals were unbalanced. The mean age of patients admitted to the study wards was 59.07 years (sd 5.89) and differed significantly between hospitals (Table 2). The CCI was the lowest in hospital five and the highest in hospital one. The latter was a tertiary referral center, which seemed compatible with the high comorbidity score and long length of stay of admitted patients. Wards had a total mean bed occupancy of 25.21 patients (sd 3.74). Mean nurse staffing levels (NHPPD) ranged from 1.75 to 4.20 across wards with a mean of 2.48 (sd 0.59). Furthermore, the mean proportion of bachelor nursing hours was 0.59 (sd 0.14). The mean crude mortality rate calculated from the database with 68 estimates (per ward and period) was 20.49 (sd 22.50) per 1000 admissions. Other outcome measures such as unexpected death, death until 72 h after CPR, and death until 72 h after unplanned ICU admission per 1000 admissions had a mean of 1.80 (sd 2.95), 0.76 (sd 1.59), and 0.62 (sd 1.33), respectively. The grand mean of the combined mortality rate was 3.18 (sd 4.09) per 1000 admissions and it ranged from zero to 19.42 between wards and across time.

**Table 1:** Number of estimates per hospital and study period (*n* = 68)

	T0	T1	T2	T3	Total estimates	Total patient records examined
Hospital 1	4	2			6	3627
Hospital 2	4	2			6	4893
Hospital 3	4	2	2		8	3628
Hospital 4	4	4	2		10	4197
Hospital 5	4	4	4		12	5665
Hospital 6	4	4	2	2	12	6030
Hospital 7	4	4	4	2	14	6227

**Table 2:** Comparison of study measures between hospitals

Hospital	1	2	3	4	5	6	7	total	*p*-value
Age	59.95 (4.06)	63.04 (5.64)	57.80 (4.67)	62.50 (4.02)	57.15 (5.55)	53.22 (5.34)	62.05 (3.80)	59.07 (5.89)	0.002
CCI	2.37 (0.68)	1.27 (0.79)	0.95 (0.47)	1.78 (1.06)	0.68 (0.52)	2.01 (1.27)	1.34 (0.86)	1.51 (1.00)	0.006
NHPPD	3.65 (0.57)	2.02 (0.29)	2.75 (0.58)	2.56 (0.14)	2.23 (0.35)	2.33 (0.27)	2.40 (0.22)	2.48 (0.59)	< 0.001
Prop. bachelor nurse hours	0.65 (0.05)	0.58 (0.12)	0.52 (0.15)	0.59 (0.10)	0.48 (0.16)	0.72 (0.12)	0.56 (0.09)	0.59 (0.14)	0.001
Nurse assistant HPPD	0.15 (0.16)	0.58 (0.17)	0.10 (0.14)	0.22 (0.19)	0.38 (0.11)	0.34 (0.16)	0.22 (0.09)	0.31 (0.21)	< 0.001
Logistic support staff HPPD	0.45 (0.11)	0.10 (0.15)	0.22 (0.17)	0.13 (0.14)	0.28 (0.13)	0.49 (0.17)	0.13 (0.05)	0.26 (0.20	< 0.001
Hospital LOS	9.80 (1.89)	7.03 (2.55)	5.97 (1.94)	6.44 (1.79)	7.74 (3.24)	7.68 (2.45)	5.45 (1.55)	7.24 (2.62)	0.030
Bed occupancy	27.88 (2.51)	23.27 (2.74)	25.73 (3.22)	25.17 (2.17)	23.53 (5.81)	25.31 (2.11)	27.37 (2.54)	25.21 (3.74)	0.100

This table compares the means including standard deviations from study measures between hospitals

*P*-values are calculated using a Kruskal Wallis test

*CCI* Charlson Comorbidity Index, *NHPPD* Nursing Hours Per Patient Day, *Prop*. Proportion, *HPPD* Hours Per Patient Day, *LOS* Length of Stay

In model 1 we calculated the estimates and 95% CI’s using an LMM adjusted for clustering and study time (Table 3, model 1). We found no significant association between NHPPD and any of the study outcome variables in this first analysis. The proportion of bachelor nursing hours (estimate − −-7.931, p 0.061), nurse assistant HPPD (estimate 1.405, p = 0.656), and logistic support staff HPPD (estimate 4.612, p = 0.145) had no significant impact on the combined mortality rate in an analysis similar to model 1 without adjustment for other confounders (LMM only adjusted for clustering and study time). Contrarily, the mean patient’s age (estimate 0.325, p = 0.005) and CCI (estimate 1.207, p = 0.036) showed a significant positive relation with the combined mortality rate (LMM only adjusted for clustering and study time). However, since it is important to adjust for the potential clinical effects of the beforementioned variables on the patient’s outcome, we included all variables in model 2. The inclusion of these variables improved our model in all analyses demonstrated by the reduction in AIC’s and BIC’s (Table 3, model 2). No relation was found between NHPPD and crude mortality, unplanned ICU admissions, death after unplanned ICU admissions, and the length of stay when properly adjusted for possible confounders. We did find a significant negative effect between NHPPD and unexpected death, CPR, death after CPR, and the combined mortality rate in model two. Additionally, we found in the latter analysis a significant negative association between the proportion nurse Bachelor hours and unexpected death and the combined mortality rate (LMM model 2 including NHPPD; unexpected death estimate -8.438, p = 0.005; combined mortality estimate -8.845, p 0.023).

**Table 3:** Linear mixed model estimates showing the effect of the mean nursing hours per patient day on study outcome measures

Dependent variables	Model 1				Model 2					
	Estimate	95% CI of est.		*p*-value	Estimate	95% CI of est.		*p*-value	Δ AIC	Δ BIC
		Lower	Upper			Lower	Upper			
Crude mortality	−3.043	−10.670	4.584	0.426	−2.458	− 10.444	5.528	0.538	−33.9	−34.1
Unexpected death	−0.848	−2.316	0.621	0.252	−1.737	−3.013	− 0.460	0.009	−32.1	− 32.4
CPR	−0.870	−2.020	0.280	0.135	−1.271	−2.454	−0.087	0.036	−15.3	−15.5
Death after CPR	−0.631	−1.363	0.100	0.089	−0.836	−1.576	−0.097	0.028	−12.4	− 12.7
Unplanned ICU admission	−0.201	−3.399	2.997	0.900	−0.063	−3.378	3.253	0.970	−30.5	−24.8
Death after unplanned ICU admission	−0.085	−0.739	0.569	0.791	0.047	−0.754	0.847	0.907	−3.2	−3.5
combined mortality rate	−1.525	−3.544	0.493	0.136	−2.771	− 4.471	−1.071	0.002	−33.9	−34.1
Hospital LOS	−0.172	− 0.955	0.612	0.661	−0.023	− 0.836	0.791	0.955	−11.5	− 11.8

Estimates of fixed effects, 95% confidence intervals and *p*-values resulting from two linear mixed models concerning the relation between the mean Nursing Hours Per Patient Day and the patient outcome measures in column one (dependent variables)

Model 1: adjusted for clustering (random effect) and study time (fixed effect)

Model 2: adjusted for clustering (random effect), study time, the proportion of nurse Bachelor hours, nurse assistant HPPD, logistic support staff HPPD, Charlson Comorbidity Index and age (fixed effects)

Δ *AIC* Akaike’s Information Criterion of model 2 - model 1, Δ *BIC* Schwarz’s Bayesian Information Criterion of model 2 - model 1

*CPR* Cardiopulmonary Resuscitation, *ICU* Intensive Care Unit, *LOS* Length of Stay (in mean days)

**Fig. 1:**
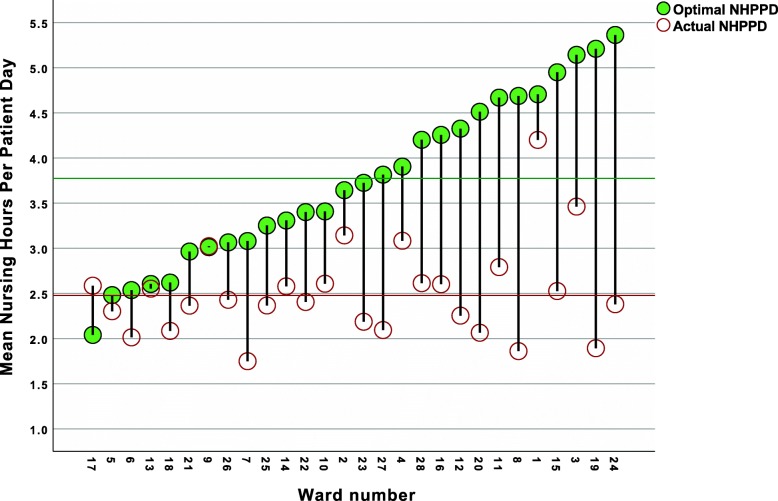
Calculated optimal mean optimal NHPPD’s versus actual NHPPD’s per study ward. The green line corresponds with the grand mean optimal NHPPD and the red line with the grand mean actual NHPPD

Using the linear regression equation resulting from model 2 with as dependent variable the combined mortality rate we calculated the theoretically optimal mean NHPPD per ward taking into account all averaged individual ward properties per period (age “a”, CCI “b”, proportion nurse Bachelor hours “c”, nurse assistant HPPD “d”, and logistic support staff HPPD “e”). The following equation was constructed considering the NHPPD estimate of −-2.771: NHPPDoptimal = (2.569 + (a*0.156) + (b*1.470) + (c*-8.845) + (d*-1.180) + (e*7.480)) / 2.771. In only one ward this resulted in a lower optimal NHPPD than the actual NHPPD (Fig. 1, ward 17) while the optimal NHPPD corresponded with the actual NHPPD in ward 9 and 13. In all other wards, the theoretically optimal NHPPD was higher than the actual available NHPPD considering all ward properties. The range of actual NHPPD’s was 1.75 (ward 7, sd 0.31) to 4.20 (ward 1, sd 0.57) and differed significantly across wards (Kruskal Wallis test, p = 0.006). The optimal NHPPD’s ranged from 2.04 (ward 17, sd 0.34) to 5.36 (ward 24, sd 0.80) and were statistically different between wards (Kruskal Wallis test, p = 0.001). The grand mean for the optimal NHPPD was 3.78 (sd 1.03) while the grand mean for the actual NHPPD was 2.48 (sd 0.59).

## Discussion

This longitudinal observational study explored the association between nurse staffing levels and adverse mortality on medical and surgical wards in Belgian hospitals. We found that wards with on average higher mean NHPPD’s had lower mean proportions of patients experiencing unexpected death, CPR and death after CPR. Furthermore, we estimated the adverse mortality by combining the mortality after CPR and after unplanned ICU admission with the unexpected death rate and found a significant negative relation between NHPPD and the combined mortality rate. These analyses included adjustment for the unbalanced data between wards, across time, and for important clinical confounders such as nurse’s education, nurse’s support staff HPPD, patient’s comorbidity and mean age. The most apparent limitation of this study was that it included a post-hoc analysis of data collected for another study investigating the effect of a Rapid Response System on patient outcomes. However, in this previous study we purposefully collected data on nurse staffing levels and patient morbidity because we hypothesised, considering the existing literature, that they could be important confounders when studying patient mortality. Moreover, we only used control group data of the previous study to avoid interference of the intervention on patient outcomes. Daily data concerning staffing and patient comorbidity were not available. Therefore, we aggregated data to the ward level resulting in staffing levels and patient comorbidity estimates per period. Since the staffing levels and patients vary from day to day on a specific ward, and because of the relatively low incidence of unexpected death, it seemed sensible to calculate estimates from partially available data collected during a long period of time. In future research however, it is advisable to collect data concerning all admitted patients and staffing levels per admission day to improve the precision of ward level data. Another limitation of this study was that we missed patients’ comorbidity scores from one hospital at two different time points because of technical difficulties in obtaining this data. Moreover, a comorbidity score does not account for complicated procedures or acute changes in the patient’s condition. Additionally, we did not include patient turnover as a possible confounder in our analysis. Therefore, we may have underestimated nurses’ experienced workload and its possible effect on outcome [20]. We did not collect ICU characteristics such as ICU medical-nursing staffing levels or ICU bed availability which could have influenced the composite mortality outcome. Since we only included Belgian hospital wards in this study, the generalisability of our results is limited. Our study is one of the few studies investigating the effect of nurse staffing levels on adverse mortality on medical and surgical wards using ward level data instead of aggregated hospital-level data [1, 8]. Evidence suggests that nurse-to-patient ratios and nurse education both influence many patient outcomes including in-hospital mortality. However, crude patient mortality has been criticized for its lack of sensitivity to nursing care [21]. This could be the case because of the possible low impact nurses have on the total (crude) mortality since some patients’ deaths are inevitable. In our previous study we showed that an important part of the crude mortality rate were patients who died after receiving a do not attempt resuscitation (DNAR) code [14]. Moreover, when we applied a new definition for unexpected death, we discovered that its incidence is much lower than anticipated and it does not correspond to the crude mortality rate. In this study we used the same outcome indicators and found that nurse staffing was not associated with the crude mortality rate, but it does have an impact on unexpected death, death after CPR and the combined mortality rate. Patient surveillance is one of the nursing activities that are frequently neglected when workload is high [5, 22]. Missed patient deterioration could result in cardiac arrest with CPR or even unexpected death. In this study we used the combined mortality rate as an estimate for adverse mortality which included unexpected death, death after CPR, and death after an unplanned admission to the ICU. This measure comprises adverse deaths and excludes the expected mortality on the general ward (i.e., death of a patient with an untreatable disease). Moreover, a patient’s death after CPR or transfer to the ICU could be the consequence of late detection of patient deterioration (missed care). Therefore, we think it is much more sensible to use the combined mortality rate, estimating adverse death, when studying the effect of nurse staffing levels. Although this was an observational study, we used longitudinal data which enabled us to take into account the interference of time. Moreover, we adjusted for clustering and other clinical confounders. This makes a causal interpretation much more plausible but certainly not definite. Because of the limited amount of multicentre studies collecting longitudinal data at the ward level, it is very difficult to transfer research results into practice [10]. In a recent study, Fagerström and colleagues collected data from 36 wards in four Finnish hospitals on a daily basis and demonstrated a relation between nurse workload and patient mortality [23]. However, they did not include the nurse’s education level or the potential influence of other team members supporting patient care. Another recent longitudinal study by Griffiths et al. (UK), also found a significant relation between low nurse staffing levels and an increased risk of death [24]. The authors of this last study included registered nurses and unregistered nursing assistant staffing levels, but they only collected data in one acute care hospital. To our knowledge, our study is therefore the first longitudinal, multicentre study including nurse staffing levels, nurse education and care team composition with data collected on the ward level. In a key European study from 2014, Aiken and colleagues showed that every 10% increase in the number of bachelor’s degree nurses was associated with a decreased likelihood of death by 7% within 30 days of admission [3]. The authors also concluded that increased nursing workload was correlated with patient mortality. We found a mean NHPPD of 2.48, consistent with the known numbers for Belgium (24 h / 10.8 patients per nurse = approximately 2.22 NHPPD) [25]. Furthermore, the proportion of nurses with a bachelor’s degree was also comparable to previous research (0.59 in this study vs. 0.55 by Aiken et al.). The mean crude mortality rate in our study was, however, much larger (2.05% in this study vs. 1.20% by Aiken et al.). The most evident explanation for this difference is that Aiken et al. only included surgical patients, which may result in lower comorbidity scores and a reduced mortality rate [26]. We confirm previous results concerning the effect of nurse staffing and nurse education level on patient mortality. Importantly, the composition of care teams is heterogeneous around the world and even across Europe. Even nursing degree levels are not uniform in the European Union [27]. Therefore, investigating the impact of the care team composition on patient outcomes in an international context could be challenging. It seems evident, considering our results and previous research, that the proportion of highly educated nurses has a significant impact on patient safety and subsequently on mortality. Hospitals and even governments could calculate the optimal proportion of nurses with a bachelor’s degree per ward using the same method as described in this study. Furthermore, the Institute Of Medicine released a report recommending that the proportion of nurses with a bachelor’s degree should be 80% by 2020 to provide safe care [28]. Our figures indicate that this is at the moment certainly not the case in Belgian hospitals (mean proportion of 59% bachelor’s degree nurses). During the last decade, researchers have been studying care processes that may prevent patients from experiencing harm during their hospital stay [29]. Since nurses have a very important role in detecting deteriorating patients on the general ward, many interventions were therefore targeted at nurses. However, the increasing care demands in acute care hospitals, combined with the nursing shortage could impact the effectiveness of such interventions [30]. Policy makers should be aware of this ongoing issue and adequate staffing levels should be determined to provide safe care [31]. Ward based minimal staffing levels should be considered relating to ward acuity measures estimating nurse’s workload and patient outcomes. Using the regression equation resulting from our analysis, we attempted to calculate the theoretically optimal NHPPD per ward. Interestingly, this allowed us to discriminate between low acuity and high acuity wards since it takes into account patient comorbidity, age and it also allows adjustment for team composition. This method could be used to calculate and adjust nurse staffing levels to a changing care environment. We compared our results with the mandatory NHPPD provided by the Australian government to provide safe care [9]. The actual mean NHPPD in Belgium is lower than the minimal mandatory NHPPD for hospital wards provided by the Australian government (1.75 in this study vs. minimal 3.00 for one-day hospitalisation including day surgery and dialysis). Furthermore, the highest optimal NHPPD calculated in this study is still lower than the minimal mandatory NHPPD for high complexity acute care wards in Australia (5.36 in this study vs. minimal 5.75 for category C non-ICU wards). This shows that Belgian acute care hospitals have comparatively low nurse staffing levels and that the calculated optimal NHPPD corresponds roughly to the Australian mandatory staffing levels.

## Conclusion

This is the first multicentre study exploring the effect between nurse staffing levels and patient mortality using longitudinal, ward level data with adjustment for patient’s age and comorbidity, nurse education level and care team composition. Our results are in accordance with previous research and confirm the association between higher nurse staffing levels and lower patient mortality. Furthermore, we also found that a higher proportion of bachelor’s degree nurses is related to a reduction in patient mortality. We proposed a new method to estimate optimal staffing levels using ward level data.

## Data Availability

The datasets used and/or analysed during the current study are available from the corresponding author on reasonable request.

## Abbreviations

AIC: Aikake information criterion
BIC: Bayesian information criterion
CCI: Charlson comorbidity index
CPR: Cardiopulmonary resuscitation
DNAR: Do not attempt resuscitation
DNR: Do not resuscitate
HPPD: Hours per patient day
ICU: Intensive care unit
LMM: Linear mixed model
LOS: Length of stay
NHPPD: Nursing hours per patient day
NPR: Nurse-to-patient ratio
PCS: Patient classification systems
SW-RCT: Stepped wedge randomised controlled trial
UK: United Kingdom
